# Older Americans With Disability Are Vulnerable to Economic and Food Insecurity During COVID-19

**DOI:** 10.1093/geroni/igab046.777

**Published:** 2021-12-17

**Authors:** Shinae Choi, Eun Ha Namkung, Deborah Carr

**Affiliations:** 1 The University of Alabama, Tuscaloosa, Alabama, United States; 2 Korea Institute for Health and Social Affairs, Sejong, Ch'ungch'ong-namdo, Republic of Korea; 3 Boston University, Boston, Massachusetts, United States

## Abstract

This study investigated whether older Americans with physical disability were vulnerable to three types of economic insecurity (difficulty paying regular bills, difficulty paying medical bills, income loss) and two types of food insecurity (economic obstacles, logistical obstacles) during the early months of the COVID-19 pandemic. We evaluated the extent to which associations are moderated by three personal characteristics (age, sex, race/ethnicity) and two pandemic-specific risk factors (job loss, COVID-19 diagnosis). Data were from a random 25 percent subsample of the Health and Retirement Study participants who completed a COVID-19 module administered in 2020. Our analytic sample included 3,166 adults aged 51 and older. We estimated logistic regression models to document the odds of experiencing each hardship. Persons with three or more functional limitations reported significantly higher odds of both types of food insecurity, and difficulty paying regular and medical bills, relative to those with no limitations. After controlling for health conditions, effects were no longer significant for paying medical bills, and attenuated yet remained statistically significant for other outcomes. Patterns did not differ significantly on the basis of the moderator variables. Older adults with more functional limitations are vulnerable to economic and food insecurity during the pandemic, potentially exacerbating the physical and emotional health threats imposed by the pandemic. Our findings reveal an urgent need to promote policies and procedures to protect older adults with disability from economic and food insecurity. Supports for older adults with disability should focus on logistical as well as financial support for ensuring food security.

